# Caspases: evolutionary aspects of their functions in vertebrates

**DOI:** 10.1111/j.1095-8649.2009.02184.x

**Published:** 2009-03

**Authors:** K Sakamaki, Y Satou

**Affiliations:** * Department of Animal Development and Physiology, Graduate School of Biostudies, Kyoto UniversityKyoto 606-8501, Japan; ‡ Department of Zoology, Graduate School of Science, Kyoto UniversityKyoto 606-8502, Japan

**Keywords:** apoptosis, caspase, evolution, inflammation, terminal differentiation, vertebrates

## Abstract

Caspases (cysteine-dependent aspartyl-specific protease) belong to a family of cysteine proteases that mediate proteolytic events indispensable for biological phenomena such as cell death and inflammation. The first caspase was identified as an executioner of apoptotic cell death in the worm *Caenorhabditis elegans*. Additionally, a large number of caspases have been identified in various animals from sponges to vertebrates. Caspases are thought to play a pivotal role in apoptosis as an evolutionarily conserved function; however, the number of caspases that can be identified is distinct for each species. This indicates that species-specific functions or diversification of physiological roles has been cultivated through caspase evolution. Furthermore, recent studies suggest that caspases are also involved in inflammation and cellular differentiation in mammals. This review highlights vertebrate caspases in their universal and divergent functions and provides insight into the physiological roles of these molecules in animals.

## Introduction

**Fig. 1:**
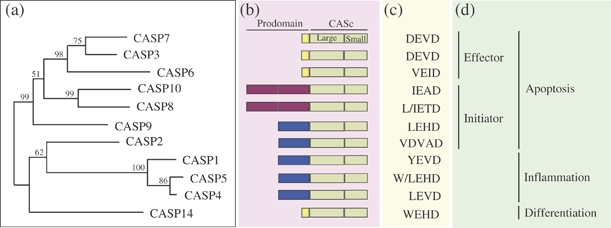
Schematic diagram of the human caspases. (a) The phylogenetic relationship of human caspases. A molecular phylogenetic tree of human caspases was generated based on the alignment of the amino acid sequences for the CASc protease domain by the maximum likelihood method. Numbers noted at the branches represent the bootstrap values obtained from 1000 replications. The gene identification numbers cited for the generation of the tree were listed in Table SI. (b) Protein structure. Procaspases carry a prodomain connected with a catalytic region (CASc) composed of large and small subunits. Caspases-3, -6, -7 and -14 contain a short prodomain (yellow), whereas the other caspases carry a long prodomain containing a caspase-recruitment domain (blue) or two death effector domains (red). (c) Substrate specificity. Preferred sequences in the substrates recognized and cleaved by each caspase were indicated as described previously (Earnshaw *et al.*, 1999; Mikolajczyk *et al.*, 2004;). (d) The physiological roles of caspases. Caspases are divided into three subfamilies in accordance with their physiological distinction between inflammatory, initiator and effector caspases. In contrast with other caspases, it is proposed that caspase-14 acts as a factor required for keratinocyte differentiation in the skin.

Caspases (cysteine-dependent aspartyl-specific protease) belong to a family of cysteine proteases that recognize certain tetrapeptide motifs and cleave after an aspartate residue in their substrates. Caspases are essential for the initiation and execution of apoptosis and for the processing and maturation of the inflammatory cytokines (Nicholson, 1999). To date, a number of caspases have been identified in various vertebrate and invertebrate species. In humans *Homo sapiens*, 11 caspases including caspases-1 to caspases-10 and caspase-14 have been identified [Fig. 1(a)]. Several additional caspases, including caspase-11, caspase-12 and caspase-13 have been detected in other mammals such as rodents and the cow *Bos taurus*. These 14 mammalian caspases are classified into several groups according to their phylogenic relationships, which are correlated with functional relatedness (Lamkanfi *et al.*, 2002). Two sub-groups of caspases are characterized as initiators (caspases-2, -8, -9 and -10) and effectors (caspases-3, -6 and -7) in the apoptotic signalling pathway [Fig. 1(d)]. As the inflammatory caspases, caspases-1, -4, -5, -11, -12 and -13 are included in one subfamily [Fig. 1(d)]. Caspase-14 is a unique caspase as it belongs to neither apoptotic caspases nor inflammatory caspases (Lippens *et al.*, 2000). Recently, caspases-15, -16, -17 and -18 were identified as new members of the caspase family in vertebrates, although their function has not yet been identified (Eckhart *et al.*, 2005; Sakata *et al.*, 2007; Eckhart *et al.*, 2008;). In addition, fish-specific caspases have been found: caspy, caspy2 and caspase-recruitment domain (CARD)-casp8 (Masumoto *et al.*, 2003; Sakamaki *et al.*, 2007;). Not limited to vertebrates, caspases have been identified in a wide variety of animals such as sponge *Geodia cydonium*, *Hydra vulgaris*, sea anemone *Aiptasia pallida*, nematode *Caenorhabditis elegans*, fruitfly *Drosophila melanogaster*, sea urchin *Strongylocentrotus purpuratus* and ascidians *Ciona intestinalis* and *Ciona savignyi* (Shaham, 1998; Cikala *et al.*, 1999; Lamkanfi *et al.*, 2002; Terajima *et al.*, 2003; Wiens *et al.*, 2003; Weill *et al.*, 2005; Dunn *et al.*, 2006; Robertson *et al.*, 2006; Kumar, 2007;). Several caspases in these organisms are orthologues to vertebrate caspases. A short form of sponge caspases shows highest sequence similarity to human caspase-3 and has caspase-3-like enzyme activity (Wiens *et al.*, 2003). Therefore, it is proposed that the function of caspases is conserved in animals through evolution. Despite conservation over time, caspases have also diverged species specifically because the number of caspases identified is different in each organism. These specific caspases may play novel roles in various species. This review summarizes information about caspases identified in vertebrates regarding their physiological roles and functions, concentrating specifically on fish and mammalian caspases. Furthermore, by comparing caspases between vertebrates and invertebrates, between mammals and non-mammals or among different mammals, the authors attempt here to understand when they came into existence during evolution and how the molecular mechanisms regulating the activation of caspases were established.

## General features and classification of caspases

### General features of caspases

Generally, caspases are synthesized as an inactive zymogen composed of a prodomain and a caspase catalytic region (CASc) [Fig. 1(b)]. Caspases-3, -6, -7, -14, -16 and -17 contain a short prodomain, whereas the other caspases carry a long prodomain that is involved in protein–protein interactions. Caspases-1, -2, -4, -5, -9, -11, -12, -13 and CARD-casp8 possess a prodomain termed a ‘caspase-recruitment domain’ (CARD), and caspases-8, -10 and -18 carry repeated motifs called the ‘death effector domain’ (DED) in the prodomain [Fig. (1b)] and caspase-15, caspy and caspy2 have a ‘pyrin domain’ (PYD) at the amino terminus. These CARD, DED and PYD show a six-helix bundle structure and are thought to mediate the assembly of large signalling complexes (Park *et al.*, 2007). For the generation of large and small subunits, caspases are auto-cleaved or processed by upstream caspases at two sites between the prodomain and the CASc and in the CASc. Finally, active caspases assemble from two large and two small subunits and recognize and cleave the specific sequence of target substrates as shown in Fig. 1(c).

### Caspases that are involved in apoptosis

#### Caspases-3, -6 and -7

The shared structural characteristic of caspases-3, -6 and -7 is a short prodomain connected with a CASc. This property suggests that they are unable to form a complex with other molecules through their prodomain. To assume the active form, other caspases must process caspases-3, -6 and -7. These three molecules mainly act as the executioners of the apoptotic process. In particular, caspase-3 is a main effector caspase that cleaves the majority of the substrates in cells undergoing apoptosis (Fischer *et al.*, 2003). Caspase-3 is processed and activated by initiator caspase-8 or caspase-9. Caspase-7 shares similarity with caspase-3 in its structure and function (Wei *et al.*, 2000; Degterev *et al.*, 2003;). Therefore, these two caspases show overlapping activity in apoptosis. The processing of poly(ADP-ribose) polymerase (PARP) is mainly dependent on caspase-7 rather than caspase-3 (Germain *et al.*, 1999). Although it shows a resemblance to caspases-3 and -7 in its protein structure, caspase-6 acts differently (Thornberry *et al.*, 1997). Although caspase-6 is also thought to act downstream of initiator caspases (Wolf & Green, 1999), substrates recognized by caspase-6 are restricted to a few proteins including lamin A (Takahashi *et al.*, 1996). Because the lack of caspase-6 function does not disturb the progression of apoptosis (Zheng *et al.*, 1999; Ruchaud *et al.*, 2002;), its unique role in the apoptotic signalling pathway is still uncertain, although it probably acts as an effector caspase.

In fishes, orthologues for caspases-3, -6 and -7 have been identified in several species such as rainbow trout *Oncorhynchus mykiss* (Walbaum), zebrafish *Danio rerio* (Hamilton), salmon *Salmo salar* L. and sea bass *Dicentrarchus labrax* L. Laing *et al.*, 2001; Yabu *et al.*, 2001; Chakraborty *et al.*, 2006; Takle *et al.*, 2006; Reis *et al.*, 2007*a*;). Uniquely, two genes encoding orthologs for mammalian caspase-3, caspase-3A and caspae-3B have been detected in zebrafish, medaka *Oryzias latipes* (Temminck & Schlegel) and stickleback *Gasterosteus aculeatus* L. (Table SI). Although the physiological role of these two fish paralogues has not yet been clarified, their expression patterns are distinct in the early developmental stages of the embryo (Iijima & Yokoyama, 2007). Caspase-3A is expressed in the tailbud, whereas caspase-3B is detected in the head. In fishes, it may be necessary to differentially induce these caspases-3A and -3B in embryos for the execution of the programme of apoptosis, which serves as a fail-safe mechanism in early development to eliminate physiologically damaged embryo. Despite the fact that the single gene encoding caspase-3 exists in the genomes of eutherian mammals, another *casp3*-like gene, *casp17*, was recently identified in monotremes, chickens and frogs (Eckhart *et al.*, 2008). Even though the function of caspase-17 has not yet been defined, there is evidence that suggests caspase-17 functions as an effector molecule – its protein structure is similar to that of caspase-3. In these animals, caspase-17 may represent a counterpart of either caspase-3A or caspase-3B in fish.

#### Caspase-2

Caspase-2 is one of the initiator caspases, which are required for the execution of apoptosis, and contains a long prodomain called CARD (Fig. 1). It has been proposed that procaspase-2 is activated in the complex formed with CARD-containing proteins through CARD–CARD interactions. Caspase-2 is critical for mitochondrial outer membrane permeabilization and the release of apoptotic factors in response to DNA-damaging agents (Zhivotovsky & Orrenius, 2005). Caspase-2 is also activated for death receptors (DRs)-mediated or heat shock-induced apoptosis as well as oocyte cell death in mammals (Bergeron *et al.*, 1998; Wagner *et al.*, 2004; Shin *et al.*, 2005; Bonzon *et al.*, 2006; Lavrik *et al.*, 2006; Tu *et al.*, 2006;). These lines of evidence suggest the importance of caspase-2 in apoptosis. However, caspase-2 is not required for thymocyte or neuronal apoptosis (O’Reilly *et al.*, 2002). It is likely that another caspase complements the role of caspase-2 in these cells. Although caspase-2 has been identified in *Danio rerio* (Inohara & Nunez, 2000) and its expression was detected in the ovary (accession number BM082390), its physiological role in fish is still unknown. In the amphibian *Xenopus laevis*, it has been reported that oocyte cell death is regulated by the modification of caspase-2 (Nutt *et al.*, 2005), suggesting the universal physiological role caspase-2 plays in oocyte cell death in vertebrates.

#### Caspases-8, -10 and -18

**Fig. 2:**
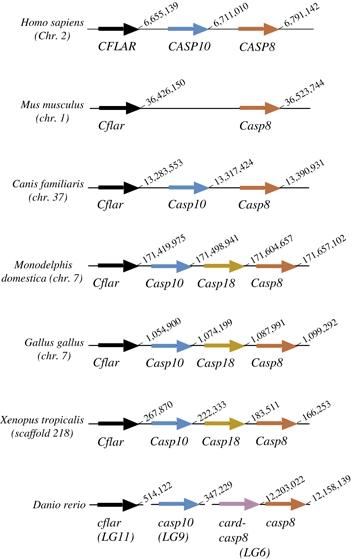
Physical map of the genomic region including the *Casp8* gene and its related genes in vertebrates. The bold arrows indicate the coding region and orientation of the gene. In humans and dogs, the *CASP8*, *CASP10* and *CFLAR* genes form a cluster on the chromosome (chr.) 2 or 37. Rodents have lost the *Casp10* gene. Opossums, chickens and frogs have the additional *Casp8/Casp10*homologous gene, *Casp18*, between the *Casp8* and the *Casp10* genes. In *Danio rerio*, the *casp8*, *casp10* and *cflar* genes localize on different chromosomes and the *card-casp8* gene exists upstream of the *casp8* gene. Numbers indicate the starting point of the coding region in the Ensemble genome database. The gene identification numbers cited for the generation of the map were listed in Table SI. The figure was generated by combining the genomic data of dogs and opossums with the data published in a previous study (Sakata *et al.*, 2007).

Caspase-8 carries tandem DED motifs in the amino-terminal prodomain and an active CASc protease domain at the carboxyl terminus (Boldin *et al.*, 1996; Muzio *et al.*, 1996; Sakamaki *et al.*, 1998; Nakajima *et al.*, 2000;). Caspase-8 is a key molecule in apoptotic induction mediated through cell surface ‘death receptors’ such as Fas (APO-1/CD95) in mammals. Like caspase-8, caspase-10 possesses two DEDs and a CASc, and its overall structure is highly similar to caspase-8 (Lamkanfi *et al.*, 2002). Although caspase-8 has an established role as an initiator of DR-mediated apoptosis, the function of caspase-10 has not yet been completely clarified (Fischer *et al.*, 2006). Despite potential overlap and redundancy, it has been shown that caspases-8 and -10 have distinct functions in human T-cell sub-sets (Wang *et al.*, 1999; Chun *et al.*, 2002;). cFlar (also termed as c-FLIP) is identified as a molecule that regulates the activation of caspase-8 (Thorburn, 2004). The cFlar protein consists of two DEDs and an inactive CASc, indicating a structure similar to caspases-8 and -10; therefore, it is hypothesized that the genes encoding these three molecules are phylogenetically related. Indeed, these three genes are contiguously aligned within the same region on human chromosome 2 (Fig. 2). Similarly, these genes in the mouse *Mus musculus*, dog *Canis familiaris*, chicken *Gallus gallus* and West African clawed frog *Xenopus tropicalis* genomes localize within a region of <120 kb on a single chromosome in each respective species (Fig. 2). The three genes form a cluster in mammals, birds and amphibians, with the exception of the absent mouse *Casp10* gene. Additionally, by comparing genome databases in vertebrates, a third *Casp8/Casp10* homologue (*casp8/10*), localized between the *casp8* and the *casp10* genes, was identified in both chickens and frogs but not in humans, mice or dogs (Sakata *et al.*, 2007). This caspase-8/10, which was tentatively named in the previous study, is now designated as caspase-18 in the GenBank database (NP_001038154). Chicken *Casp18* has a genomic structure similar to *Casp8*, *Casp10* and *Cflar* (Sakata *et al.*, 2007). Furthermore, the analysis of the marsupial and monotreme genomes shows that the *Casp18* gene exists in both of these basally diverging mammalian lineages (Fig. 2 and data not shown). In the genome of the opossum *Monodelphis domestica*, the *Casp18* gene localizes between the *Casp8* and the *Casp10* genes; this discovery was confirmed in a recent report by Eckhart *et al.* (2008). It appears that the *Casp18* gene was ancestral but was deleted from the genome when placental mammals first appeared.

In contrast to vertebrates, only one gene similar to *casp8* was identified in both fruitfly *D. melanogaster* and ascidian *C. intestinalis* genomes (Chen *et al.*, 1998; Terajima *et al.*, 2003;). Based on phylogenetic analysis, the extant ascidian gene product appears to represent an ancestral chordate form of vertebrate caspase-8, caspase-10, caspase-18 and cFlar (Sakata *et al.*, 2007). This suggestion leads to a hypothesis that the last common ancestor of ascidians and vertebrates had a single ancestral ‘*casp8*’ gene, and, after the divergence of ascidians and vertebrates, this ‘*casp8*’ gene experienced tandem duplication events and the divergence of products into four genes (*casp8*, *casp10*, *casp18* and *cflar*) in vertebrates (Sakata *et al.*, 2007). In the additional study, the authors observed that the sea urchin *Paracentrotus lividus* caspase-8 (accession number EU078681) has a protein structure more similar to ascidian caspase-8 than vertebrate caspase-8. In the fish lineage, the *casp18* gene localizing close to the *casp8* gene is not detectable in any fish genome databases. Instead of the *casp18* gene, another gene, *card-casp8*, is localized close to the *casp8* gene (Fig. 2). This replacement suggests the possibility that the ancient ‘*casp8*’ gene was locally duplicated to become both *casp8* and *card-casp8*, which might occur once in the fish lineage. As an alternative explanation, the ancient *casp18* gene modified the genome structure and exchanged with the *card-casp8* gene by gene conversion. Card-casp8 carries a CARD, but not two DED motifs, in the N-terminal prodomain. That is, the *card-casp8* gene probably represents a fish substitute for the *casp18* gene identified in other vertebrates. As a result of a genome duplication event followed by gene arrangements that occurred in teleost lineages (Postlethwait, 2007), the *casp10* and *cflar* genes segregated from the locus harbouring the *casp8* gene (Fig. 2 and Sakata *et al.*, 2007). Caspase-8 and CARD-casp8 isolated from *Danio rerio* and *O. latipes* have a pro-apoptotic ability (Eimon *et al.*, 2006; Sakamaki *et al.*, 2007; Sakata *et al.*, 2007;). In addition, in the Japanese flounder *Paralichthys olivaceus* (Temminck & Schlegel), caspase-10 was identified as a pro-apoptotic molecule (Kurobe *et al.*, 2007). Consequently, caspase-8 and its related molecules conserve their pro-apoptotic activity in vertebrates.

#### Caspase-9

Caspase-9 was isolated as an initiator caspase containing a CARD motif at the N-terminus. This caspase plays an essential role in the mitochondria-mediated cell death pathway (the intrinsic pathway) (Li *et al.*, 1997), and it generates the active effector caspases through the cascade. Therefore, caspase-9 deficiency leads to impaired processing and subsequent activation of the downstream caspases (Hakem *et al.*, 1998; Kuida *et al.*, 1998;). An adaptor molecule (Apaf-1) is necessary to form a complex with caspase-9 through their homophilic CARD–CARD interactions, leading to the auto-processing and activation of caspase-9 in the assembly. Because the database of the sea urchin *S. purpuratus* contains both caspase-9 and Apaf-1 (accession numbers XP_799258 and XP_796156), the machinery required for the intrinsic apoptotic pathway seems to be conserved within the deuterostomes. In bony fish, caspase-9 has been identified and characterized in *D. labrax*, and both increased expression and protease activity of caspase-9 were detected in the head kidney of *D. labrax* infected with *Photobacterium damselae* ssp. *piscicida*, suggesting the involvement in the advanced septicaemia (Reis *et al.*, 2007*b*).

### Inflammatory caspases

#### Caspase-1

Caspsae-1 was previously known as the interleukin (IL)-1β-converting enzyme (ICE). This molecule processes the inactive prointerleukin-1β (proIL-1β) to produce the active cytokine IL-1β, which is a major mediator of inflammation in mammals (Cerretti *et al.*, 1992; Thornberry *et al.*, 1992;). Active caspase-1 also cleaves other cytokines: IL-18, IL-1F7b and IL-33 (Nadiri *et al.*, 2006). The activation of caspase-1, which has a CARD in the prodomain, occurs in the cytosolic complex termed ‘inflammasome’ through CARD–CARD interactions (Martinon *et al.*, 2002). A previous study proposed that ‘pyroptosis’ is a form of cell death induced by pathogen distinct from apoptosis and necrosis (Cookson & Brennan, 2001). Additionally, a recent report confirmed that pyroptosis is induced through caspase-1 activation (Fernandes-Alnemri *et al.*, 2007). Thus, caspase-1 is crucial for the maturation of inflammatory cytokines in inflammation and may play a role as a pro-apoptotic molecule under pathologically adverse conditions. In the gilthead seabream *Sparus aurata* L., caspase-1 has been identified as an inflammatory caspase in fish lineage (Lopez-Castejon *et al.*, 2008). Caspase-1 was expressed in the immune tissues in *S. aurata* adults, but whether this molecule has the ability to process proIL-1β and proIL-18 or if it is involved in pyroptosis has not yet been clarified. Caspase-1 is also present in chickens and *Xenopus* (Table SI), suggesting the possibility that this molecule is the major effector in inflammation in all vertebrates.

#### Caspases-4, -5, -11, -12 and -13

**Fig. 3:**
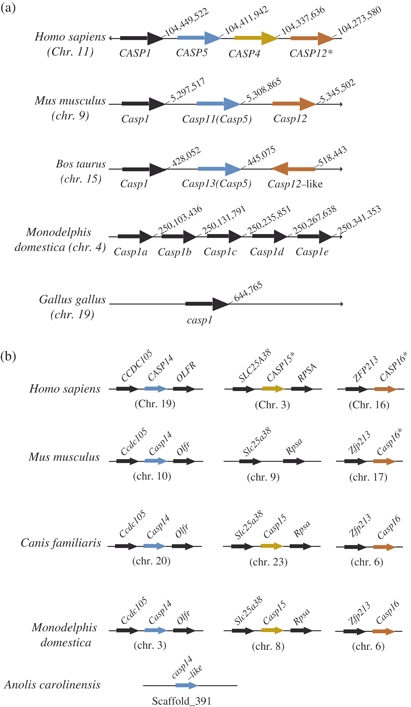
Chromosomal analyses of the *Casp1* and *Casp14* genes and their orthologues. (a) Physical map of the region including the *Casp1* gene and its related genes in vertebrates. The bold arrows indicate the coding region and orientation of the gene. In humans, the *CASP1*, *CASP5*, *CASP4* and *CASP12* genes form a cluster on the chromosome 11. The *Casp1*, *Casp11(Casp5)* and *Casp12* genes and the *Casp1*, *Casp13(Casp5)* and *Casp12*-like genes form a cluster on the genome of the mouse and cow, respectively. Five *Casp1*-like genes were detectable in the opossum genome database. (b) Physical map of the region including the *Casp14*, *Casp15* or *Casp16* genes in vertebrates. The bold arrows indicate the coding region and orientation of the gene. In humans, dogs and opossums, three genes were identified in the genome, whereas rodents have lost the *Casp15* gene. In the anole lizard *Anolis carolinensis* genome, the *casp14*-like gene was identified. *Ccdc105*, coiled-coil domain containing 105; *Olfr*, olfactory receptor; *Slc25a38*, solute carrier family 25, member 38; *Rpsa*, ribosomal protein SA; *Zfp213*, zinc finger protein 213. Asterisks indicate the presence of non-functional caspase sequences. Identification numbers of the genes examined were listed in Table SI.

In addition to caspase-1, caspases-4, -5, -11 -12 and -13 belong to a subfamily of caspases called ‘inflammatory caspases’ in mammals. These molecules have a similar prodomain (CARD) as caspase-1. Caspases-4 and -5, which are primarily identified in humans, show a high degree of similarity in their protein structure. As primates such as the macaque monkey also possess both *CASP4* and *CASP5* genes (Table SI), it is thought that these two genes arose by tandem duplication of the ancestral *CASP4/5* gene after the divergence of primates and other mammals. Additionally, mouse caspase-11 and cow caspase-13 are orthologues of primate caspases-4 and -5. In a strict sense, these caspases are thought to be a counterpart of caspase-5 but not caspase-4 (Lin *et al.*, 2000). The genes for caspases-4, -5, -11 and -13 form a cluster with the *CASP1* gene on the chromosome in mammals. They are arranged as *CASP1*, *CASP5*, *CASP4* and *CASP12* in humans; *Casp1*, *Casp11(Casp5)* and *Casp12* in mice and *Casp1*, *Casp13(Casp5)* and *Casp12*-like in cows [Fig. 3(a)]. In contrast, there are no additional genes closely related to this group except the *casp1* in birds, amphibians and fishes [Fig. 3(a) and data not shown]. Therefore, it is proposed that gene amplification occurred in the early stages of evolution to mammals. This inference is supported by a genome database search, indicating that the *Casp1* gene and *Casp1*-like genes localize in the same region of the genome in the opossum and platypus [Fig. 3(a) and data not shown].

Human caspase-4 but not caspase-5 directly processes proIL-1β, proIL-18 and proIL-1F7b, although its protease activity is not high compared with caspase-1 (Lin *et al.*, 2000; Kumar *et al.*, 2002;). Mouse caspase-11, which is orthologous to human caspase-5, has the capacity to cleave caspases-1 (Kang *et al.*, 2000), and investigations with *casp11*-deficient mice confirmed processing activity (Wang *et al.*, 1998). These mutant mice failed to produce IL-1β and are resistant to endotoxic shock induced by bacterial endotoxins, showing a similar phenotype to *casp1*-deficient mice. Therefore, it was proposed that caspase-11 co-operatively works with caspase-1 and might be an upstream activator of caspase-1. Similarly, human caspase-5 forms a complex with caspase-1 and leads to caspase-1 activation (Martinon *et al.*, 2002). For human caspases-4 and -5 and mouse caspase-11, specific substrates identified are vanishingly few except for caspase-1; however, they are able to cleave caspase-3 (Kamada *et al.*, 1997; Schwartz *et al.*, 1999; Lin *et al.*, 2000;). This suggests a possibility that they are involved in apoptotic processes. Indeed, human caspase-4 works as a pro-apoptotic molecule (Kamada *et al.*, 1997). Although it may be too early to conclude, it appears not only caspase-1 but also caspases-4, -5 and -11 may be involved in inflammatory cell death (see below).

The gene encoding caspase-12 is localized together with other genes for the inflammatory caspases in the same chromosomal region. As shown in Fig. 3(a), they are contiguously aligned with no unrelated genes between. However, the function of caspase-12 is thought to be distinct from that of other inflammatory caspases because it has been shown that mouse caspase-12 is involved in endoplasmic reticulum (ER) stress-induced apoptosis (Nakagawa *et al.*, 2000). Caspase-12-deficient mouse embryonic fibroblasts were resistant to apoptosis induced by genotoxic stimuli. As a special point of note: this capability of caspase-12 is not always conserved in mammals. For example, human caspase-12 is expressed as a truncated protein in the major population because of polymorphism (Fischer *et al.*, 2002). Considering that non-human primates carry the intact *Casp12* gene in the genome, it has been concluded that human caspase-12 has been varied to a non-functional molecule through ER stress-induced apoptosis over the course of evolution.

#### Caspy and caspy2

**Fig. 4:**
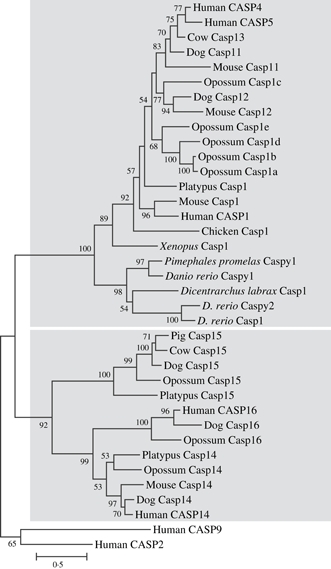
The phylogenetic relationship of vertebrate caspases. A molecular phylogenetic tree of vertebrate caspases (caspase-1, caspase-14 and their relatives) was generated based on the alignment of the amino acid sequences for the CASc protease domain by the maximum likelihood method. Human caspases-2 and -9 were used as the outgroup proteins for rooting the tree. Numbers noted at the branches represent the bootstrap values are shown only when >50%. Identification numbers of caspases examined were listed in Table SI.

In fishes, *casp1* has been isolated *S. aurata* (Lopez-Castejon *et al.*, 2008) and is also detectable in several fish genome databases. However, none of other inflammatory caspases discovered in mammals are yet identified in fish. As supporting data, the authors found that the genomes of the *D. rerio*, *O. latipes* and *G. aculeatus* lack cluster with caspases-1 and other caspases. Interestingly, two caspases, caspy and caspy2, whose CASc protease domains show similarity to that of caspase-1 (Fig. 4), have been identified in *D. rerio* (Masumoto *et al.*, 2003). Caspy and caspy2 carry a PYD in the prodomain instead of the typical CARD of inflammatory caspases. The *caspy* gene is also detected in the database of fathead minnow *Pimephales promelas* Rafinesque (accession number DT175611) but the *caspy2* gene is undetectable in other fish databases. Caspy2 might be a derivative of caspy in *D. rerio*, although they exhibit different substrate specificity (Masumoto *et al.*, 2003). Furthermore, caspy, but not caspy2, is involved in morphogenesis of the jaw and gill-bearing arches of fish larvae (Masumoto *et al.*, 2003). Caspy is also able to interact with the *D. rerio* orthologue of apoptosis-associated speck-like protein containing a CARD (ASC), which is characterized as an adaptor molecule forming a complex with caspase-1 (Srinivasula *et al.*, 2002). Therefore, it is necessary to further analyse whether caspy and caspy2 are inflammatory caspases in fish.

### Caspases required for terminal differentiation

#### Caspase-14

Caspase-14 has a small prodomain similar to that of caspases-3, -6 and -7 (Fig. 1). Previously, the function of this molecule had remained elusive compared with other caspases because it was thought not to be involved in apoptosis and inflammation. One of the main reasons that caspase-14 plays no role in these processes is that the expression is restricted almost exclusively to the suprabasal layers of the epidermis and the hair follicles (Lippens *et al.*, 2000). Because the proteolytic activation of caspase-14 was associated with stratum corneum formation, it was suggested that this caspase may play a pivotal role in keratinocyte differentiation (Lippens *et al.*, 2000). A recent study concerning the phenotype of caspase-14-deficient mice showed that caspase-14 works in the processing of filaggrin proteins and functions in the protection against water loss and ultraviolet (UVB) photodamage (Denecker *et al.*, 2007). Therefore, caspase-14 is involved in protection from noxious environmental conditions. Meanwhile, caspase-14 is shown to be associated with large prodomain caspases, including caspases-1, -2, -4, -8 and -10 (Hu *et al.*, 1998) and is processed *in vitro* by active caspase-8 and caspase-10 and *in vivo* after ligation of the DR (Ahmad *et al.*, 1998). The possibility still remains that caspase-14 is involved in cell death in the very restricted tissue or some unknown rare cell type.

### Caspases with undefined physiological roles

#### CARD-casp8

**Fig. 5:**
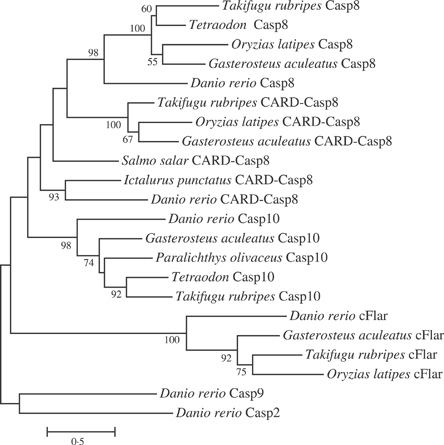
The phylogenetic relationship of fish caspase-8 and its orthologues. A molecular phylogenetic tree of fish caspases and cFlar was generated based on the alignment of the amino acid sequences for the CASc protease domain by the maximum likelihood method. *Danio rerio* caspases-2 and -9 were used as the outgroup proteins for rooting the tree. Identification numbers of molecules examined were listed in Table SI

The CASc of CARD-casp8 is most similar to that of caspase-8 (Sakata *et al.*, 2007). At first, the cDNA for CARD-casp8 was identified in the channel catfish *Ictalurus punctatus* (Rafinesque) and characterized as fish caspase-8 (Long *et al.*, 2004). However, the *I. punctatus* molecule has a CARD but lacks two DED motifs at the N-terminus. CARD-casp8 is found in *D. rerio*, *O. latipes*, *G. aculeatus*, *S. salar* and fugu *Takifugu rubripes* (Temminck & Schlegel) and forms a single clade with caspase-8, caspase-10 and cFlar in a phylogenetic tree (Fig. 5). The authors found that the gene coding CARD-casp8 is localized to the upstream of the *casp8* gene in the genomes of the *D. rerio*, *O. latipes*, *G. aculeatus* and *T. rubripes* (Fig. 2 and Sakamaki *et al.*, 2007; Sakata *et al.*, 2007;). Unexpectedly, this gene, which is tentatively termed as *card-casp8* in the research, has not been detected in any other vertebrates, suggesting that it is a fish-specific gene. As described above, it is thought that the ancestral ‘*casp8*’ gene has diverged into *casp8* and *casp8*-like genes by duplication and over the course of fish evolution, an ancestral *casp8*-like gene has replaced its DED with a CARD motif (Sakata *et al.*, 2007). The *casp8*-like gene might acquire a new function with pro-apoptotic activity as described previously (Sakamaki *et al.*, 2007).

#### Caspases-15 and -16

Caspase-15 was recently added as the new member to the caspase family list (Eckhart *et al.*, 2005). This molecule is unique among mammalian caspases because of its prodomain predicting to fold into a PYD. Based on phylogenetic analysis, the CASc of caspase-15 is most similar to that of caspase-14 (Fig. 4). This suggests the possibility of the involvement of caspase-15 in the caspase-14-mediated signalling pathway, although data in support of this have not yet appeared. Probably, the activation of caspase-15 occurs in the complex with PYD-containing proteins through PYD–PYD interactions. Because caspase-15 is absent from or inactive in some mammalian species including mice and humans [Fig. 3(b)] (Eckhart *et al.*, 2006), it is difficult to understand the universal role of caspase-15 in animals.

The existence of another *CASP14*-like gene in humans has been suggested in a previous report (Centola *et al.*, 1998). Although the mRNA for the *CASP14*-like gene was detected in the database (accession number AF098666), it is difficult to understand whether gene products are functional because it is impossible to predict the open reading frame. That is, this gene might be a pseudogene in humans [Fig. 3(b)]. Among opossum *Trichosurus vulpecula*, cDNA clones published in the GenBank database, two cESTs (accession numbers DY593381 and EG596106) contain sequences similar to human *CASP14*-like (data not shown). By assembling opossum cDNA sequences, it is possible to predict one open reading frame consisting of 300 amino acids. In a different species of opossum (*M. domestica*), a similar partial cDNA has been already identified as caspase-16 (accession number ABP93399). The predicted CASc domain in opossum caspase-16 is more similar to that of caspase-14 than other caspases. By searching the public genome databases, the authors identified *Casp16* in the mouse, dog and platypus [Fig. 3(b) and Table SI). *Casp16* clearly localizes close to the *Zfp213* gene in the animals examined, suggesting the conservation of synteny of the *Casp16* gene-containing genomic region in mammals. By molecular phylogenetical analysis, it is clear that caspases-14, -15 and -16 form a single clade in a phylogenetic tree (Fig. 4). In contrast, non-mammalian vertebrates including *D. rerio*, *Xenopus* and chickens appear to lack these three genes [Fig. 3(b) and Eckhart *et al.*, 2006]. In the reptile *Anolis carolinensis*, only the *casp14*-like gene is detectable in the preliminary genome database [Fig. 3(b) and Eckhart *et al.*, 2008]. Therefore, *Casp14*, *Casp15* and *Casp16* might have diverged from an ancestral gene during the evolution of mammals.

## Functions and physiological roles of caspases

### Apoptosis

The major function of caspases is to mediate cell death (apoptosis). Caspase-mediated apoptosis follows two main pathways, one extrinsic and the other intrinsic or mitochondrial-mediated. The extrinsic pathway is triggered by stimulation of various cell surface receptors on cells. The activated receptors transmit apoptotic signals to the intracellular complex with an initiator caspase, caspase-8. The subsequent activation of caspase-8 initiates the ‘caspase cascade’ to activate downstream effector caspases, involving caspases-3, -6 and -7.

The intrinsic pathway arises from signals that originate within the cell as a consequence of cellular stress or DNA damage. The stimulation or inhibition of different Bcl-2 family members results in the leakage of cytochrome *c* from mitochondria, and the formation of the assembly composed of cytochrome *c*, Apaf1 and caspase-9. The subsequent activation of caspase-9 initiates the caspase cascade. At the end of the cascade, effector caspases cleave a wide variety of signal transduction proteins, cytoskeletal and nuclear proteins, chromatin-modifying proteins, DNA repair proteins and endonucleases, finally leading to cell death.

#### The extrinsic pathway

The apoptotic signalling pathway designated as the extrinsic pathway is mediated through cell surface receptors called ‘death receptors’. So far, five DRs, tumour necrosis factor type-I receptor (TNFR1), Fas, DR3 and receptors 1 and 2 for TNF-related apoptosis-inducing ligand (TRAIL-R1 and TRAIL-R2) have been isolated in mammals and they contain a ‘death domain’ (DD) in the cytoplasmic region (Aggarwal, 2003). Among these receptors, Fas has been the best characterized (Ashkenazi & Dixit, 1998; Nagata, 1999; Thorburn, 2004;). Oligomerization of Fas by its ligand recruits the adaptor molecule FADD, which carries each DED and DD, to the cytosolic domain of Fas through homophilic interactions mediated by the DDs. Procaspase-8, which is an inactive form, associates in turn with FADD by interactions between their DEDs (Boldin *et al.*, 1996; Muzio *et al.*, 1996;). Within the Fas–FADD–procaspase–8 complex, called the ‘death-inducing signalling complex’ (DISC) (Kischkel *et al.*, 1995), procaspase-8 undergoes auto-cleavage to convert to an active form. Through cleavage, activated caspase-8 activates downstream effector caspases and Bid, a member of the Bcl-2 family, eventually leading to cell death (Stennicke *et al.*, 1998; Chou *et al.*, 1999; McDonnell *et al.*, 1999; Lavrik *et al.*, 2003;). Cells deficient in caspase-8 fail to undergo Fas-mediated apoptosis (Juo *et al.*, 1998; Kawahara *et al.*, 1998; Varfolomeev *et al.*, 1998;). Apoptotic signals induced by TNFR1 and TRAIL-Rs also require caspase-8 in a similar fashion (Thorburn, 2004). Thus, an initiator caspase-8 is indispensable for the induction of apoptosis downstream of multiple different DRs in mammals.

Recently, two DRs, which are designated as the *D. rerio* haematopoietic DR and the ovarian TNFR, have been identified and characterized in *D. rerio* (Long *et al.*, 2000; Bobe & Goetz, 2001;). By further analysis, it has now been confirmed that they are fish orthologues corresponding to mammalian TRAIL-Rs (Eimon *et al.*, 2006). In addition, other components (death ligands, TNFR1, Fas, FADD, caspase-8 and bid) required for the extrinsic pathway were also identified in *D. rerio* (Inohara & Nunez, 2000; Coultas *et al.*, 2002; Long *et al.*, 2004; Eimon *et al.*, 2006; Kratz *et al.*, 2006; Sakata *et al.*, 2007;). Clearly, the machinery for apoptotic signal transduction has been possessed in teleosts including *D. rerio*. To support this tentative theory, the authors also confirmed that orthologues of Fas, FADD and caspase-8, which were identified in *O. latipes*, have ability as a pro-apoptotic molecule (Sakamaki *et al.*, 2007). These three molecules are able to induce apoptosis and substitute for the functions of their mammalian counterparts in mammalian cells. These lines of evidence indicate that the apoptotic machinery is conserved in vertebrates. One note of importance: the prototype of DRs carrying a DD has not yet been identified in flies and ascidians (Chen *et al.*, 1998; Terajima *et al.*, 2003;). This suggests that the extrinsic apoptotic pathway was established in early vertebrate evolution.

#### Intrinsic signalling pathway

The intrinsic-dependent or mitochondria-dependent pathway is triggered in a wide variety of apoptotic stimuli, including ultraviolet irradiation, steroids, chemotherapeutic drugs and growth factor deprivation. Both pro-apoptotic and anti-apoptotic members of the Bcl-2 family regulate the intrinsic pathway (Gross *et al.*, 1999). In the forward progression of this pathway, apoptotic signals lead to cytochrome *c* release from mitochondria following the formation of the complex consisting of cytochrome *c*, caspase-9, Apaf-1 and ATP. In the assembly of a heptameric complex termed as the ‘apoptosome’, caspase-9 is activated, released to cytosol and then cleaves the downstream caspases including caspases-3 and -7. Apaf-1 plays a role as an adaptor indispensable for the formation of apoptosome and is a platform mediating caspase-9 activation. Deficiency of *Apaf-1* as well as *Casp9* in mouse embryonic fibroblasts resulted in the resistance to apoptotic stimuli such as etoposide (Yoshida, 2003). In addition to cytochrome *c*, several pro-apoptotic factors such as Smac and apoptosis-inducing factor are also released from mitochondria during apoptosis and implicated in various aspects of the cell death process (Jin & El-Deiry, 2005).

#### ER stress-induced apoptosis

ER stress, which is induced by the accumulation of unfolded or malfolded proteins and the disruption of calcium homeostasis, leads to cell death (Breckenridge *et al.*, 2003; Momoi, 2004;). Caspase-12 was thought to play a role in this process because caspase-12 is localized in the ER and caspase-12-deficient mice were resistant to ER stress-induced apoptosis (Nakagawa *et al.*, 2000). In humans, caspase-12 is not involved in this process as described above. In place of caspase-12, it has been reported that caspase-4 compensates in ER stress-induced cell death in human cells (Hitomi *et al.*, 2004). A recent study has provided further analysis of the involvement of mouse caspases-12 and human caspase-4 in the induction of ER stress-induced apoptosis (Obeng & Boise, 2005). At the present, therefore, it is not clear whether both caspases-12 and -4 are involved in ER stress-induced apoptosis. Because *CASP1*, *CASP5* and *Casp11* are adjacent to *Casp12* or *CASP4* in the genome [Fig. 3(a)] and work as inflammatory caspases, both caspases may have a similar function in inflammation. This inference was supported by a report showing that human caspase-12 plays a role in inflammation and innate immunity (Saleh *et al.*, 2004). In ER stress-induced apoptotic process, it is certain that at least the activation of caspases-3 and -9 through the intrinsic pathway is still required (Obeng & Boise, 2005; Masud *et al.*, 2007;).

#### DNA-damaged cell death

When cells are inactivated by DNA damage, they undergo apoptotic cell death (Roos & Kaina, 2006). Apoptosis induced by many chemical genotoxins is thought to be a consequence of the blockage of DNA replication. This abnormality is detected by checkpoint molecules, which signal the downstream protein p53. The p53 protein then induces transcriptional activation of pro-apoptotic factors such as Fas and Puma and Bax, which are pro-apoptotic members of the Bcl-2 family, resulting in cell death (Roos & Kaina, 2006). Caspase-2 is known as the only procaspase constitutively present in the nucleus (Zhivotovsky *et al.*, 1999) and activated by DNA-damaging agents; therefore, caspase-2 is also involved in DNA-damaged cell death. Recently, it has been shown that the activation of caspase-2 occurs in a complex with RAIDD and PIDD when cells are stimulated by genotoxic stress (Tinel & Tschopp, 2004). RAIDD is an adaptor protein carrying both CARD and DD that binds to caspase-2 through their homophilic CARD–CARD interactions (Chou *et al.*, 1998). PIDD containing a DD is induced by p53 and required for the formation of the complex (Tinel & Tschopp, 2004). This trimolecular complex consisting of caspase-2, RAIDD and PIDD is now designated as ‘PIDDsome’. That is, the PIDDsome is formed when cells are exposed to genotoxic stress. This complex is a platform for caspase-2 activation that is the same as DISC and apoptosome required for the activation of caspase-8 and caspase-9.

### Inflammation

Caspase-1 is a representative member of a subclass of caspases involved in inflammation because it has been well characterized as ICE. The gene encoding caspase-1 exists in all vertebrates (Fig. 4). Caspase-1 cleaves proforms of cytokines, IL-1β and IL-18, which are potent mediators of inflammation that stimulate fever, recruitment and activation of immune cells and the production of secondary cytokines (Dinarello, 1998). To process these cytokines, caspase-1 must be activated in a complex called inflammasome in advance. For the formation of this complex, ASC, which carries both CARD and PYD motifs, plays an important role as an adaptor and associates with caspase-1 through CARD–CARD interactions. Furthermore, several types of inflammasomes have been identified in recent studies. For the assembly of an inflammasome, members of the NOD-leucine-rich repeat (NLR) family including NALPs, NAIP, IPAF and Cardinal are required and act as regulators for caspase-1 (Martinon & Tschopp, 2007). These NLR proteins oligomerize with caspase-1 either directly or through ASC in response to inflammatory triggers or bacterial invasion. Different inflammasomes are activated by different signals. By infection of *Staphylococcus aureus*, NALP3, which is a member of the NLR family, forms the inflammasome with caspase-1, ASC and Cardinal and leads to the activation of caspase-1. In the presence of anthrax lethal toxins, another NLR family member, NALP1, forms a complex with caspase-1, caspase-5 and ASC and results in the activation of the caspases. In mice, it has been demonstrated that caspase-11 makes a complex with and is required for the activation of caspase-1 (Wang *et al.*, 1998). At the present, it is not clear whether this complex contains members of the NLR family. As mouse caspase-11 is thought to be a counterpart of human caspase-5 (Martinon *et al.*, 2002), it is likely that both caspases-1 and -11 form an inflammasome with ASC and NALP1 in mice. Interestingly, it has been reported that caspase-12 derived from humans of African descent blocks the caspase-1 catalysis, suggesting that it functions as a negative regulator of inflammatory caspase activation (Saleh *et al.*, 2004). In fishes, only ASC has been identified (Masumoto *et al.*, 2003; Sun *et al.*, 2008;), but NALP molecules required for the formation of inflammasomes have not been detected in fish genome databases (Stein *et al.*, 2007). Meanwhile, the large groups of novel, fish-specific NLR proteins are found and highly conserved in each species (Stein *et al.*, 2007). By expanding lineage-specific gene families that are involved in pathogen recognition, therefore, fish might accomplish the host defence system distinct from mammals.

IL-1β orthologues have been cloned in chickens (Weining *et al.*, 1998), the amphibian *X. laevis* (Zou *et al.*, 2000) and in many species of bony fish such as *O. mykiss*, carp *Cyprinus carpio* L. and *D. labrax* (Zou *et al.*, 1999; Fujiki *et al.*, 2000; Scapigliati *et al.*, 2001;). IL-18 is also detected in teleost species (Huising *et al.*, 2004). It appears these cytokines act in inflammation in non-mammals. However, there is no evidence indicating the processing by caspase-1 in their biological activity. Based on the absence of the critical aspartic acid residue in IL-1β in *O. mykiss*, *Xenopus* and chicken, it has been proposed that the cleavage of proIL-1β by caspase-1 that has been established in mammals is a recent phenomenon (Huising *et al.*, 2004). If this inference is true, it is necessary to find the specific physiological role of caspase-1 in non-mammals. This role may soon be revealed; however, the novel function of caspase-1 involved in the secretion of proIL-1α, which is a functional analogue of proIL-1β, was recently found in mammals (Keller *et al.*, 2008). Caspase-1 directly interacted with and promoted secretion of proIL-1α without processing. At this point, therefore, one cannot rule out the possibility that caspase-1 functions in the maturation and secretion of inflammatory cytokines even in non-mammals.

The activation of caspase-1 not only leads to inflammation but also causes an inflammatory form of cell death in certain pathological conditions in mammals. This type of cell death is designated as ‘pyroptosis’ (Cookson & Brennan, 2001). Pyroptosis occurs in response to infection by a number of intracellular bacterial and viral pathogens and is also observed in cells treated with non-infectious agents like lipopolysaccharide (LPS) (Fernandes-Alnemri *et al.*, 2007). In cells stimulated with several inflammatory factors, the complex containing caspase-1 and ASC, which is termed as ‘pyroptosome’, is formed and activates caspase-1, resulting in pyroptosis and the release of the proinflammatory cytokines (Fernandes-Alnemri *et al.*, 2007). Whether caspase-1 and ASC form an assembly as an inflammasome or a pyroptosome by inflammatory stimuli seems to depend on the associate proteins. In the case of the pyroptosome, both Pyrin containing a PYD motif and PSTPIP1 (proline–serine–threonine phosphatase interaction protein 1) are required for the formation of the complex (Yu *et al.*, 2007). In any case, a molecular platform is necessary to recruit and activate caspase-1.

### Differentiation

Recent studies indicate that caspase activation does not always lead to cell death or inflammation, but it might be important for terminal cell differentiation or cell-fate determination instead. Several reports have presented evidence for these expanded roles of caspases (Abraham & Shaham, 2004; Kuranaga & Miura, 2007; Lamkanfi *et al.*, 2007;). Caspase-14, which has been identified without any definitive connection to apoptosis and inflammation, is now shown to contribute to keratinocyte differentiation through processing of filaggrin proteins (Denecker *et al.*, 2007). Interestingly, it has also been suggested that caspase-14 is involved in ‘cornification’. Cornification is type of cell death distinct from apoptosis and has been observed as a mammalian-specific phenomenon in the skin. A similar phenomenon, lens formation in the eye, is the result of retention of the lens fibre cell integrity following degradation of mitochondria and other organelles. In this process, caspase-like protease activity was detected (Morozov & Wawrousek, 2006). That is, the activation of caspases seems to be associated with terminal differentiation of the skin and lens. Even in caspases such as caspase-8, which is defined as a pro-apoptotic molecule, it has been proposed that they are involved in non-apoptotic events. In humans, caspase-8 is shown to regulate lymphocyte proliferation in both a positive and a negative manner. Patients with homozygous mutations in the *CASP8* gene show defects in their activation of lymphocytes (Chun *et al.*, 2002). Furthermore, caspase-8 contributes to cell migration and adhesion (Helfer *et al.*, 2006). In the fruitfly, Dredd, a *Drosophila* orthologue of caspase-8, triggers NF-κB activation by directly processing NF-κB, suggesting its involvement in innate immunity (Kuranaga & Miura, 2007). In the recent study, it has been demonstrated that protease activity of caspase-8 is required for the activation of NF-κB even in human lymphocytes (Su *et al.*, 2005). Caspase-3 has also been reported as a regulator required for the differentiation of neural cells, myoblasts and osteoblasts (Kuranaga & Miura, 2007). To put it briefly, these caspases activated by strong signals cause apoptosis, whereas the local or restricted activation of caspases might be involved in non-apoptotic events. During evolution, it is likely that caspases acquired multiple capabilities to control their unique signalling pathways for both apoptotic and non-apoptotic processes.

## Conclusion

This review has attempted to briefly overview the functions and physiological roles of vertebrate caspases. Caspases involved in apoptosis are clearly identified in all vertebrates, suggesting the conserved function of caspases through evolution as executioners of cell death. However, the diversification of caspases has been steadily occurring during evolution. Currently, 16 caspases in mammals, seven in flies and four in worms have been identified, and there appears to be an increasing number of caspases with increasing evolutionary complexity as described previously (Lamkanfi *et al.*, 2002; Eckhart *et al.*, 2008;). This contention does not always hold true when one focuses on an individual caspase that has been identified in mammals and then is compared with those of non-mammalian vertebrates. Although the initiator caspase, caspase-8, and its related molecules, caspase-10, caspase-18 and cFlar, have multiplied from the ancestral single caspase-8 in early vertebrates, after their evolution to mammals, the *Casp18* gene in eutherian mammals and the *Casp10* gene in rodents have been deleted. As a result, a different phenotype is observed in each organism when caspase-8 has been inactivated. In mice, caspase-8 deficiency results in embryos that are embryonic lethal, whereas loss of caspase-8 is compatible with development in humans (Varfolomeev *et al.*, 1998; Chun *et al.*, 2002; Sakamaki *et al.*, 2002;). It is thought that either caspase-8 compensates for the role of caspase-10 in mice or that caspase-10 is no longer necessary for mice in life. Similarly, caspase-1-like molecules (caspases-4, -5, -11, -12 and -13) are detected only in mammals. Caspases-15 and -16 are also identified in mammals. Some of these caspases have disappeared or serve no function in certain animals even though they are expressed. Consequently, the redundancy and compensation of caspases are observed in mammals and are expected to occur in other vertebrates in the same way.

Two questions were posed at the beginning of this review: (1) when did caspases come into existence during evolution and (2) how were the molecular mechanisms regulating the activation of caspases established. In reference to the first query, an appropriate answer is found by comparing the genome databases of caspases that have been identified in a wide variety of animals in this review. The authors confirmed that one sub-group of caspases was newly established in the fish lineage and two sub-groups were organized in the mammalian lineage during evolution. To the second question, the authors can see a principle for the mechanism activating initiator caspases that have a long prodomain for interaction with other molecules. For the activation of initiator caspases, it is essential to make a complex such as ‘DISC’, ‘apoptosome’, ‘PIDDsome’ or ‘inflammasome’ with adaptor molecules. In contrast, effector caspases such as caspase-3 are usually activated by processing mediated through the upstream caspases. When effector caspases work as non-apoptotic functions, however, the mechanism activating them is not clear. Similarly, there is no information with regard to what causes caspase-14 activation. With this review, it was insufficient to encompass the full range of global issues in regards to caspases. In addition, a number of important issues and controversies still remain unresolved in caspase biology. One of high interest is when the first member of the caspase family came into existence in which animal during evolution. In yeast, although the presence of the caspase-like activity in cell death was suggested (Vachova & Palkova, 2007), there is no caspase gene in the genome. However, several caspases have been already identified in lower metazoan organisms such as sponges (Wiens *et al.*, 2003). In multicellular organism, caspase might come along to spoil certain cell(s) from a cell group, eventually leading to cell death. Hereafter, a comprehensive study using various organisms will be necessary to better understand caspase evolution. In particular, fishes (including both cartilaginous and bony fishes) will be key organisms in providing a new understanding of the importance of caspases; in addition, they will aid in defining the points in caspase activity that are not yet fully understood, leading to a better understanding of the evolutionary aspects of caspase biology in vertebrates.

## Supporting Information

Table SI. List of animals and their sequence data cited for the generation of physical maps and phylogenetic trees.
