# Supplementary material for: Caspases: evolutionary aspects of their functions in vertebrates
Source: J Fish Biol. 2009 Mar;74(4):727–53. doi: 10.1111/j.1095-8649.2009.02184.x (PMC2779465; doi:10.1111/j.1095-8649.2009.02184.x)
Supplement: Supplementary file 1 [file jfb0074-0727-SD1.doc]

**Table SI** List of animals and their sequence data cited for the generation of physical maps and phylogenetic trees.

**Caspases Animals**

Human Mouse Dog Opossum Platypus Other animals

Casp1 P29466 NP_033937 ENSMODP00000018459* XP_001509500

ENSMODP00000018463*

ENSMODP00000034020*

XP_001381578

XP_001381580

Casp2 P42575 NP_031636 XP_539853 XP_001366069 XP_001506482

Casp3 P42574 NP_033940 NP_001003042 NP_001033059 ENSOANP00000000517*

ABB96113

CASP4 P49662 - - - - Macaque:XP_001100287

CASP5 P51878 - - - - Macaque:XP_001100375

Casp6 P55212 NP_033941 XP_545022 EC348353 ENSOANP00000005811*

Casp7 P55210 NP_031637 XP_544026 ENSMODP00000012376* ENSOANP00000002509*

Casp8 O15519 NP_033942 NP_001041494 ENSMODP00000036244* XP_001513568

Casp9 P55211 NP_056548 NP_001026803 XP_001377659 XP_001520982

Casp10 Q92851 - XP_545593 ENSMODP00000036251* XP_001521249

Casp11 - NP_031635 Q9MZV7

Casp12 NR_000035** NP_033938 NP_001070704

Casp13 - - - - - Cow:O75601

Casp14 P31944 NP_033939 XP_853064 NP_001087242 XP_001510017 Anole lizard:GENSCAN00000101872*

Casp15 -** - XP_542720 NP_001035238 XP_001514938 Cow:NP_001029681, Pig:NP_001032224

Casp16 XP_113871 XP_997016 XP_853312 DY593381/EG596106 XP_001515957

Casp17 - - - Contig_43250

Casp18 - - - NP_001107832 XP_001517665

Cflar AAH01602 AAH29223 XP_545592 ENSMODP00000018873* XP_001507915

Chicken *X.tropicalis* Medaka Stickleback Zebrafish Other fishes

Casp1 AAC69917 ENSXETP00000017024* ENSORLP00000007938* ENSGACP00000013201* ENSDARP00000059407* Sea bass:ABB05054

Casp2 Q98943 ENSXETP00000015203* ENSORLP00000015114* ENSGACP00000013740* NP_001036160

Casp3 NP_990056 ENSXETP00000003217* ENSORLP00000010794* ENSGACP00000024816* NP_001018443

ENSORLP00000010095* ENSGACP00000024812* NP_001041531

ENSGACP00000024809*

ENSGACP00000022402*

Casp6 NP_990057 NP_001011068 ENSORLP00000002965* ENSGACP00000023057* ENSDARP00000041413*

ENSORLP00000003198* ENSDARP00000050929*

ENSDARP00000093958*

Casp7 XP_421764 NP_001016299 ENSORLP00000005993* ENSGACP00000008657* NP_001018443

ENSXETP00000041240*

Casp8 NP_989923 350965^#^ AAS91704 ENSGACP00000016969* AAS91705 Fugu:SINFRUP00000179400*

Tetraodon:GSTENP00015090001*

Casp9 AAL23701 ENSXETP00000014928* ENSORLP00000003208* ENSGACP00000007379* NP_001007405

Casp10 XP_421936 NP_001015715 ENSGACP00000005794* ENSDARP00000024255* Fugu:SINFRUP00000131803*

Hirame:BAE98150

Casp17 ABX89982 NP_001037933

Casp18 NP_001038154 DT426909 - - -

Caspy - - NP_5715800 Minnow:DT175611

Caspy2 - - NP_690840

CARD- - - ABQ42565 ENSGACP00000016983* NP_001077331 Fugu:SINFRUP00000182829*

Casp8 Catfish:AAT37512

Salmon:EG872162/CK880643

Cflar XP_421935 AAI23994 ENSORLP00000020363* ENSGACP8246 AAI16571 Fugu:SINFRUP00000145303*

Sequence data indicted by single asterisks (*) and sharps (^#^) were published in the Ensembl and JGI genome databases and the other sequence data were published in the GenBank database. Double asterisks (**) indicate the inactive gene caused by the polymorphism.
